# Perioperative Brain Function Monitoring with Electroencephalography in Horses Anesthetized with Multimodal Balanced Anesthetic Protocol Subjected to Surgeries

**DOI:** 10.3390/ani12202851

**Published:** 2022-10-20

**Authors:** Carla Murillo, Hsin-Yi Weng, Ann B. Weil, Matthias Kreuzer, Jeff C. Ko

**Affiliations:** 1 Department of Veterinary Clinical Sciences, College of Veterinary Medicine, Purdue University, West Lafayette, IN 47907, USA; 2 Department of Comparative Pathobiology, College of Veterinary Medicine, Purdue University, West Lafayette, IN 47907, USA; 3 Matthias Kreuzer, Department of Anesthesiology and Intensive Care, Technical University of Munich, School of Medicine, 81675 München, Germany

**Keywords:** electroencephalogram, EEG indices, multimodal anesthesia, horses, burst suppression, xylazine

## Abstract

**Simple Summary:**

This study aimed to investigate the use of electroencephalography (EEG) and EEG-derived (processed) indices for detecting brain activity changes perioperatively in 12 anesthetized adult horses subjected to various surgery. Frontal electrodes together with Sedline/Root monitor were used on these horses from soon after anesthesia induction and continued until the horse first attempted to stand in recovery. The EEG waves were characterized by low-frequency high amplitude alpha, theta, and alpha waves during the isoflurane maintenance and surgery, which is commonly observed in profound anesthesia. The processed EEG indices including Patient State Index, Burst Suppression Ratio, and 95% Spectral Edge Frequency changed significantly between the stages (induction, surgery, and recovery) of anesthesia. Collectively, the presence of the slow EEG wave activities and the presence of burst suppression implies that these horses were profoundly unconscious during the anesthesia. We concluded that the use of EEG in conjunction with traditional cardiorespiratory monitoring provides clinically relevant information about perioperative brain state changes in the anesthetized horses.

**Abstract:**

This study aimed to investigate the use of electroencephalography (EEG) for detecting brain activity changes perioperatively in anesthetized horses subjected to surgery. Twelve adult horses undergoing various surgeries were evaluated after premedication with xylazine and butorphanol, induction with ketamine, midazolam, and guaifenesin, and maintenance with isoflurane. The frontal EEG electrodes were placed after the horse was intubated and mechanically ventilated. The EEG data were collected continuously from Stage (S)1—transition from induction to isoflurane maintenance, S2—during surgery, S3—early recovery before xylazine sedation (0.2 mg kg IV), and S4—recovery after xylazine sedation. The Patient State Index (PSI), (Burst) Suppression Ratio (SR), and 95% Spectral Edge Frequency (SEF_95_) were compared across the stages. The PSI was lowest in S2 (20.8 ± 2.6) and increased to 30.0 ± 27.7 (*p* = 0.005) in S3. The SR increased from S1 (5.5 ± 10.7%) to S3 (32.7 ± 33.8%, *p* = 0.0001). The spectral power analysis showed that S3 had a significantly higher content of delta wave activity (0.1–4 Hz) in the EEG and lower relative power in the 3 Hz to 15 Hz range when compared to S1 and S2. A similar result was observed in S4, but the lower power was in a narrower range, from 3 Hz to 7 Hz, which indicate profound central nervous system depression potentiated by xylazine, despite the cessation of isoflurane anesthesia. We concluded that the use of EEG provides clinically relevant information about perioperative brain state changes of the isoflurane-anesthetized horse.

## 1. Introduction

General anesthesia can be accomplished by single or multiple classes of anesthetics to achieve antinociception, amnesia, akinesia, and unconsciousness [1]. Modern equine anesthetic practice employs multiple anesthetic drug classes in the development of a multimodal balanced anesthetic protocol. This may include premedication with an alpha-2 sedative agent plus an opioid, induction with a benzodiazepine together with a dissociative anesthetic, and maintenance with an inhalant agent. The welfare of the equine patient while undergoing anesthesia is maintained with subsequent monitoring of the four anesthetic components by assessing hemodynamic parameters, eye reflexes, and body movements [2,3,4,5,6]. The degree of muscle relaxation is usually judged by the anal and extremity muscle tone. The end-tidal inhalant anesthetic concentration is used to determine the depth of anesthesia changes over time.

While these monitoring parameters have been used for many years, equine anesthesia monitoring presents a unique challenge for the anesthetist. Unlike human and small animal anesthesia, the heart and respiratory rates rarely change dramatically in the anesthetized horse subjected to surgery. The respiration is usually mechanically controlled, which makes respiratory rate or character difficult to use as an indicator of the level of anesthesia. This leaves the anesthetist to rely on other surrogate signs of the central nervous system changes such as blood pressure, eye and swallowing reflexes, and muscle/anal tone for assessing the depth of anesthesia [6]. Furthermore, the amnesia and unconsciousness of the anesthetized horse are assumed based on the clinical assumption that if a horse is not moving when subjected to noxious stimuli, it must be unconscious and have no memory of such an experience. This assumption remains a mystery to us, since we cannot know this without monitoring the brain state directly and continuously in real time. To make matters worse, the equine patient may be purposefully anesthetized in a deeper plane of anesthesia than necessary in order to prevent sudden movements of the horse and to avoid the animal injuring itself or surgical personnel during a surgical procedure [7].

Numerous human and animal studies [8,9,10,11,12,13,14,15,16,17,18,19,20,21,22] have shown that general anesthetic drugs induce systematic changes in EEG patterns in a dose-dependent manner. For example, during a light plane of anesthesia, the EEG is dominated by high frequency and low amplitude beta waves (14–30 Hz). When the anesthetic level increases, the beta waves disappear and the patterns shift to low frequency and high amplitude alpha (8–13 Hz), theta (4–8 Hz), and delta (0.5–4 Hz) waves. These waves remain dominant in a moderate to deep plane of anesthesia, with alpha waves eventually becoming smaller in amplitude [10,14,15,18,23,24,25,26,27,28,29,30]. As the depth of anesthesia continues to deepen, burst suppression, an EEG pattern characterized by periods of isoelectric activity alternating with occasional high-frequency brain activity, appears [17,31,32]. In an excessive anesthetic depth, the frequency of the suppression lengthens between the bursts of brain activity, and this frequency is measured by the EEG monitor as the burst suppression ratio (SR). As the anesthetic plane continues to deepen, cortical silence occurs with a resultant quasi-isoelectric EEG pattern [10,33]. As a result, monitoring EEG provides a direct method of monitoring the brain activity associated with the change in the depth of anesthesia in humans and animals [10]. The routine use of EEG for monitoring brain function has not gained significant acceptance in equine anesthesia. Although the use of EEG for monitoring the depth of anesthesia in horses has been previously attempted with bispectral index monitoring [7,11,16,18,20,26,34,35,36], the results were not optimum, and were at times confusing.

With recent advancements in brain function monitoring technology and with a better understanding of the pharmacology of the molecular target of the anesthetic drugs on the neural circuitry, more advanced EEG monitors, including Masimo Sedline ^®^ are commercially available for use in human patients [10]. The Sedline^®^ monitor (Masimo Corporation, Irvine, CA, USA) obtains four frontal EEG channels of the raw (or unprocessed) EEG electrical signal, and provides a quantitative analysis of the brain’s electrical activity based on the changes in the various EEG power of the δ, θ, α, and β-frequency bands, to yield a single numerical value called PSI. These advanced EEG monitors utilize both unprocessed EEG and EEG-derived (processed) indices to indicate the levels of unconsciousness and, hence, the depth of anesthesia in anesthetized humans. Unfortunately, in human EEG studies, most of the information is obtained from a single drug classification, such as propofol alone, inhalant anesthetic alone, dexmedetomidine alone, or, at the most, a combination of no more than a few pharmacologic classes in a single protocol [10,28,30,37,38]. The EEG information obtained in such a situation is likely quite different from the multimodal anesthetic protocols used in horses. Therefore, it is unknown whether the use of Sedline^®^ monitors can reproduce those results observed in human studies. The use of Sedline^®^ monitors in horses and pigs has been recently explored [22,34,35]. Exploring a routine and easy-to-use continuous EEG monitoring system as an adjunct to the current cardiorespiratory monitoring in equine anesthesia is a worthwhile endeavor in order to improve the anesthetic safety in a species that has a high risk of morbidity and mortality while undergoing and recovering from general anesthesia.

The goals of this study were to (1) explore the clinical use of EEG in the multimodal anesthetic protocol to evaluate brain state changes in anesthetized horses perioperatively, and (2) to determine if any correlations exist between the EEG indices and the traditional monitoring parameters, including cardiorespiratory values, subjective anesthetic depth scores, and end-tidal isoflurane concentrations. We hypothesized that the EEG could be used continuously in monitoring equine anesthesia perioperatively to provide information about brain state changes, and the EEG parameters would be unlikely to correlate well with the traditional monitoring parameters.

## 2. Materials and Methods

This study was approved by the Animal Care and Use Committee at Purdue University. Owner consent forms were obtained before the study.

### 2.1. Animals

Twelve client-owned adult horses for soft tissue (n = 6) or orthopedic (n = 6) related surgeries that were not performed on the head were enrolled in this study. All animals were deemed healthy and free from central nervous system dysfunction and systemic illness. The aged of the horses ranged from one to fourteen years old, and the horses weighed between 317 and 522 kg (Table 1). The sexes of the horses were two intact males, three females, and seven geldings. The details of the horse information, types of surgeries, and anesthesia durations are presented in Table 2.

### 2.2. Anesthetic Protocol

All horses received an identical anesthetic protocol. The horses were premedicated with intravenous xylazine (0.44 mg/kg) and butorphanol (0.02 mg/kg) for sedation. Five to eight minutes later, the horses were induced with midazolam (0.11 mg/kg) and ketamine (2.22 mg/kg). Then, 200–300 mL of 5% guaifenesin was given to facilitate jaw tone relaxation and endotracheal intubation. Following endotracheal intubation, the horses were then maintained on isoflurane in 100% oxygen in preparation for the surgery. The same dose of butorphanol was given by IV hourly for antinociception.

### 2.3. EEG Electrode Modification and Placement

The Sedline^®^ adult adhesive forehead electrodes (L1, L2, R1, R2, ground CB, and reference CT) were each connected with a regular neurology EEG needle electrode (Neuroline subdermal electrodes, 12 × 0.4 mm- 0.5-inch × 27 gauge) using an alligator clip. This connection allowed flexibility in fitting the electrodes to different horse head sizes, and provided more precise localization of electrodes without being restricted by the size of the original adhesive electrodes. After the connection, the electrical signals and impedance were automatically screened by the Sedline^®^ monitor. The acceptable range for electrode impedance values was 0.0 to 65.0 kilo-ohms [39]. This range of electrode signals and impedance was accepted by the Sedline^®^ monitor, as indicated by the green icons shown on top of the screen for each electrode status throughout the study [39]. If an electrode was rejected by the monitor, as shown in the icon colors (red, blue, or grey), it was replaced and reexamined until accepted by the monitor.

**Figure 1:**
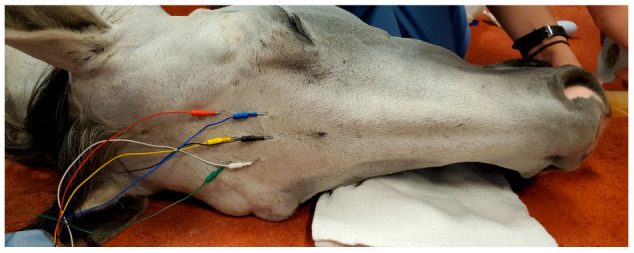
The needle electrodes were placed on the forehead of a horse soon after anesthetic induction. The needle electrode wires were color-coded and corresponded to the Sedline^®^ electrode R1 (white color wire), positioned at an approximation of the Fp2, R2 (green color wire), positioned between F4–F8, L1 (blue color wire), positioned at an approximation of the Fp1, and L2 (red color wire), positioned between F3 and F7. The CB—Ground (black color wire) and the CT—Reference (yellow color wire) electrodes were placed on the mid-sagittal line in the central and the caudal position, respectively.

**Figure 2:**
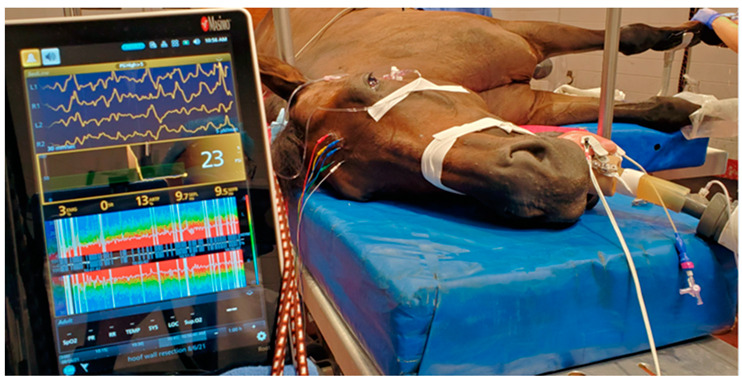
A Sedline^®^ monitor with needle electrodes connected to the forehead of a horse anesthetized with isoflurane while being prepared for surgery. The Sedline^®^ monitor shows a PSI of 23, the trend of the PSI over the last 20 min, and the 4-channel raw EEG waveforms at the top of the monitor. The SEF_95_ on the left hemisphere is 9.7 Hz and 9.5 Hz on the right hemisphere. The suppression ratio is zero, and the artifact % is 3.

The positions of the needle electrodes were similar to those described in Drewnowska et al. [34], with the position of the electrode corresponding to the position of the human EEG 10–20 system, the Sedline^®^ electrode R1 positioned at the Fp2, R2 positioned between F4–F8, L1 positioned at the Fp1, and L2 positioned between F3 and F7. The ground (CB) and the reference (CT) electrodes were placed on the mid-sagittal line in the central and the caudal position, respectively (see Figure 1). The placement of the subdermal needle electrodes was simple, and did not require hair shaving or any adhesive to secure the needle in place (Figure 2). The needle electrode stayed in position throughout the study, unless the monitor indicated a bad signal for a given electrode, then the electrode was adjusted or replaced, as mentioned previously. Alcohol or conduction gel was applied to the needle electrodes as needed to enhance conductivity as part of the adjustment.

### 2.4. EEG and Cardiorespiratory Data Collections

For each procedure, the EEG electrodes were placed as soon as (within 5 min) the horse was endotracheally intubated. The standard vital cardiorespiratory monitoring was instrumented using side-stream capnography for measuring end-tidal isoflurane and CO2, pulse oximetry for hemoglobin saturation, electrocardiogram, direct arterial blood pressure, and body temperature (esophageal and/or rectal temperature when accessible). The anesthetized horse was immediately placed on a ventilator for controlled ventilation. During the surgical preparation and surgery, mean arterial blood pressure was maintained between 65–90 mmHg using balanced electrolyte fluids and a dobutamine infusion to effect. The end-tidal isoflurane concentration was used to guide the adjustment of the isoflurane vaporizer setting based on the clinical signs of the anesthetic depth assessment. The depth of anesthesia was subjectively scored according to the criteria set in Appendix (Table A1). End-tidal CO_2_ was maintained between 35–45 mmHg, and hemoglobin oxygen saturation measured by pulse oximeter was maintained above 98%. Blood gas samples were measured at least once every hour in order to validate the non-invasive cardiorespiratory monitoring values until the horse was moved to the recovery stall.

The EEG data were collected continuously, except when the horse was transported from the induction area to the surgery room, and from the surgery room to the recovery stall. During the re-location of the horse, the EEG cable was disconnected from the monitor and reconnected once the relocation was completed. The cardiorespiratory data were collected at 5-min intervals during the anesthetic procedure until the horse entered the recovery stall. For the sake of data analysis, the anesthesia was arbitrarily divided into four anesthetic stages. Stage 1—(S1, induction stage) was the transition from intravenous injectable anesthetic induction to isoflurane maintenance while the horse was surgically scrubbed and prepared for surgery. Stage 2—(S2, surgical stage) was during the surgery and Stage 3—(S3, early recovery stage) was when the horse was discontinued from isoflurane maintenance and moved to the recovery stall for recovery, but before a dose of xylazine (0.2 mg kg IV) was administered for sedation. Stage 4—(S4, late recovery stage) was the recovery period after the xylazine sedation and until the horse started to have the first spontaneous movement with an attempt to stand.

The recorded EEG data were automatically stored by the Sedline^®^ and retrieved via the Masimo^®^ Trace^TM^ program as CSV files. The CSV files contained processed EEG indices, including high resolution (every two seconds) values of the Patient State Index (PSI, 0—total cortical silence, 100—completely awake state), % (Burst) Suppression Ratio (SR), % electromyography (EMG), 95% of the Spectral Edge Frequency (SEF_95_) on the left and right hemispheres, and % of the artifact (ART). The raw EEG data were downloaded as .edf files for visual inspection and reconstruction of a spectrogram (see below). The 2-second EEG data were further aggregated as the mean values at each 5-min interval. For the SEF_95_ data, the left and right sides of the hemisphere were calculated separately and compared using the paired t-tests. The PSI, SR, SEF_95_, EMG, and ART were compared across the stages using random-effect linear models, followed by pairwise comparisons with the Bonferroni adjustment. Both mean (± SD) and median (range) for the EEG indices were reported.

In addition, we also evaluated the raw EEG waveforms and reconstructed a density spectral array (DSA) by extracting the raw EEG traces from the recorded .edf files using MATLAB R2019 (The MathWorks, Inc. Natick, MA, USA). Because of the known technical issues with the Sedline^®^ EEG export [40], we down-sampled every recording to 89 Hz and only considered the 2nd channels displayed centrally (R1), as the other channels displayed at the top and the bottom were at higher risk to be affected by clipping. We then performed an artifact subspace reconstruction with the functions from the EEGLAB toolbox (Swartz Center for Computational Neuroscience, Institute for Neural Computation, University of California San Diego, La Jolla, CA 92093-0961, USA) [41], and the burst criterion was set to 20 as recommended [42]. We then calculated the DSA using the Welch functions with default settings and NFFT set to 256 (i.e., a frequency resolution set to 0.35 Hz). The DSA was derived from 10 s EEG segments with a 9 s overlap.

Due to the known technical issues, we only presented results regarding the relative power [40]. We normalized the power to the cumulative power in the 0.35 to 29.56 Hz range. We then extracted the DSA segments corresponding to each animal at each stage and used the median of the spectral power for further analysis. In order to statistically compare the spectral power, we calculated the area under the receiver operator characteristics curve (AUC) and 10,000-fold bootstrapped 95% confidence intervals (CI) using the MATLAB-based MES toolbox [43] for each frequency. A 95% CI exclusive of AUC = 0.5 indicated a significant difference [43]. In order to reduce the risk of false positives, we only defined a significant difference (*p* < 0.05) if at least two neighboring frequencies showed significant differences. Similar procedures have been applied previously [23,44].

## 3. Results

**Figure 3:**
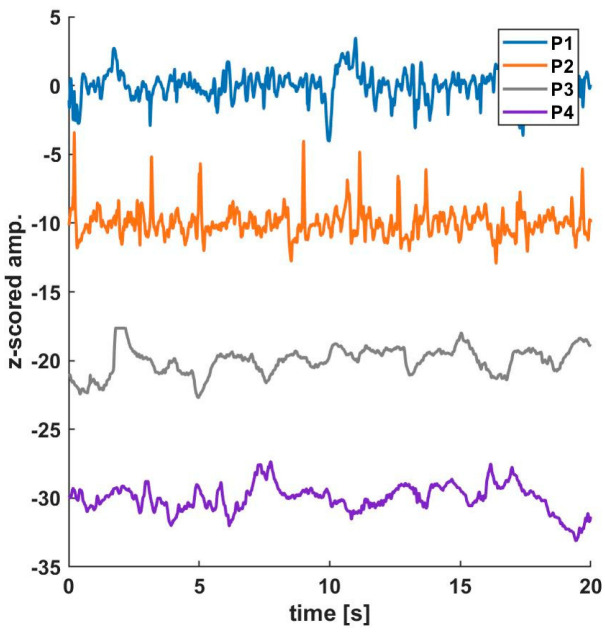
An exemplary EEG raw trace of all four stages was taken from a horse undergoing soft tissue surgery (horse #4).

**Figure 4:**
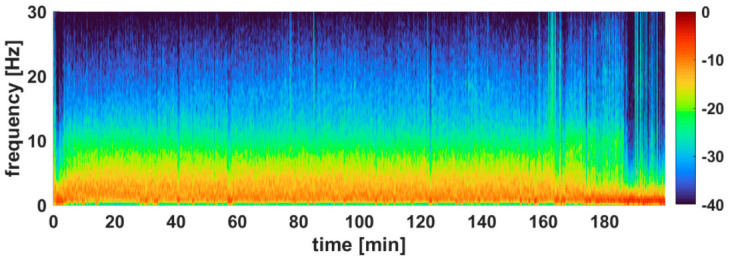
A reconstructed Density Spectral Array (DSA) example of a horse (soft-tissue surgery horse #4) is shown. The DSA was constructed with 5 s EEG segments from the entire anesthetic procedure. The artifact noise was removed using artifact subspace reconstruction. The DSA shows dominant slow wave activity as represented by the warm colors in the DSA. Towards the end of the DSA, it shifted toward cooler colors in the higher frequencies, and an increase in the power in the slower frequencies can be seen. All DSAs looked similar in the study horses.

A picture of a study horse with needle electrodes connected to the forehead and EEG recorded by the Sedline^®^ monitor during S1 is shown in Figure 2. An example of an EEG raw tracing taken from all four stages of a horse undergoing soft tissue surgery is presented in Figure 3. A reconstructed DSA example of a horse (soft-tissue surgery horse #4) is shown in Figure 4.

The processed EEG data are shown in Table 3. The mean PSI and SR values were significantly different between stages (Table 3). The mean PSI value during S2 was significantly lower than the other stages, whereas the mean SR spiked during S3 and S4. There was no significant difference between the left and right hemispheres in SEF_95_, and both were lower in S3 and S4 compared with S1 and S2. The EMG activities were significantly lower during S1 and S2 and increased significantly in S3 and S4.

The subjective depth of anesthesia score was not significantly different between stages. The mean value of the subjective depth score for S1 was 2.4 ± 0.8, with a median value of 2 (2–3), 2.5 ± 0.7 with a median value of 3 (2–3) for S2, and 2.0 ± 1.0 with a median value of 2 (1–3) for S3. There was no significant difference between the left and right hemispheres in SEF_95_, and both were lower in S3 and S4 compared with S1 and S2. The EMG activities were significantly lower during S1 and S2, and increased significantly in S3 and S4. The correlations between the processed EEG indices and subjective anesthetic depth score were all weak (Spearman’s rho ranged from 0.001 to 0.26). The subjective score in S4 was not evaluated since the horses were extubated and were given xylazine.

The mean (SD) and median (range) of end-tidal isoflurane measurements are presented in Table 3. The correlations between the process EEG parameters and the end-tidal isoflurane concentration were also weak in S1 (Spearman’s rho ranged from −0.01 to 0.16) and were slightly stronger in S2 (Spearman’s rho ranged from −0.10 to 0.36). During the general anesthetic period, the blood pressure was maintained with mean arterial blood pressure above 65 mmHg, end-tidal CO_2_ between 35–45 mmHg, hemoglobin saturation for oxygen of > 98%, and body temperature of the horse above 37 degrees Celsius.

**Figure 5:**
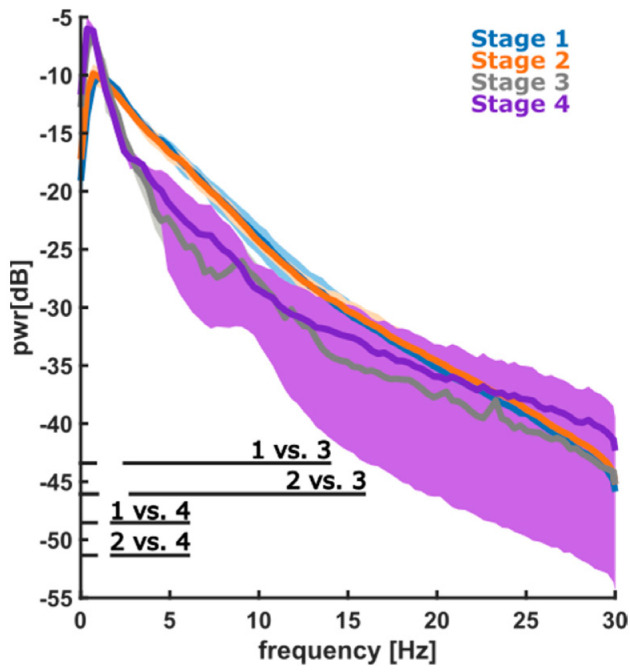
The corresponding power spectral density (PSD) plots of the 12 horses are shown. Stage 1—induction, stage 2—surgery, stage 3—early recovery, and stage 4—late recovery. The significant difference (*p* < 0.05) between stages was noted in the graph.

The spectral power analysis showed that S3 had a significantly higher content of low delta wave activity (0.1–4 Hz) in the EEG and lower relative power in the ~ 3 Hz to 15 Hz range when compared to S1 and S2. A similar result was observed in S4, but the lower power was in a narrower range, from ~3 Hz to 7 Hz. The corresponding power spectral density (PSD) plots are shown in Figure 5.

## 4. Discussion

The results of this study showed that the EEG could be used perioperatively to obtain brain state changes in a multimodal balanced anesthetic protocol for anesthetized horses.

The modern EEG monitor not only provides raw EEG waveforms, but also provides processed EEG in the form of spectrograms as well as processed EEG indices. In the current study, PSI and SR provided detailed information about the brain state changes over the stages of anesthesia. The PSI values were initially higher in the S1 and then decreased during the S2, whereas SR increased slightly from S1 to S2. The PSI scale on the Sedline^®^ monitor is between 0 (total cortical silence) and 100 (fully awake). In the anesthetized human, the PSI values are targeted between 25–50 during the surgery, which serves to indicate loss of consciousness and an optimal anesthetic plane for surgery [45,46]. The PSI values during the S1 and S2 were lower than this range, assuming the horse has the same optimal PSI range as humans, indicating that the anesthetic plane was deeper than “the optimal anesthetic plane.” The optimal anesthetic plane PSI range in the anesthetized horse is not known, however the presence of burst suppression could potentially indicate that the anesthetic plane was likely in a profound stage.

Burst suppression consists of episodes of isoelectric EEG silence alternating with high EEG voltage periods and bursts of slow waves. The SR is calculated by the Sedline^®^ monitor as the percentage (0–100) of epochs in the last 63 s where the EEG silent periods are longer than 0.5 s, and during which the EEG voltage does not exceed approximately +5 to − 5 μV [47]. One of the most common reasons to have a high burst suppression ratio under general anesthesia is an excessive depth of anesthesia [31,48,49,50,51]. The SR was present during the S1 and S2. The low PSI and presence of SR indicated that the brain activities of these horses were profoundly depressed during the transition from injectable anesthesia to inhalant anesthesia (S1) and during the isoflurane maintenance (S2).

Clinically, it is not uncommon in equine practice to have the horse anesthetized deeper than “just adequately” (i.e., no movement) [19] because having an under-anesthetized horse moving during the surgery could result in patient and personnel injury if strong spontaneous movement occurs and is not able to be quickly controlled. In the current study, the correlations between subjective depth of anesthesia score and PSI or SR were relatively weak. The subjective anesthetic depth score indicated that the depth of anesthesia during S2 was in the “medium” stage, and was “medium-deep” in S1 and S3, which suggested that these horses were in a deeper plane of anesthesia during these times than during the surgery. The subjective depth score, however, could not sufficiently quantify how much deeper the anesthesia of these horses was in S1 and S3 when compared with S2. With the PSI and SR values, we were able to quantitatively assess the changes in the cortical activity of these horses during the various stages of the anesthesia.

Several factors determine the clinical usefulness of an EEG monitor, including its ability to detect the brain state changes of a patient anesthetized with multimodal anesthetic protocols, and the response or lack of such a response to nociceptive stimuli, hence the conscious state of the anesthetized patient. Ultimately, the EEG parameters provided by the monitor are used to assess the depth of anesthesia/hypnosis and to allow titration of the anesthetic effect, in order to avoid excessive anesthesia causing central nervous system depression or inadequate anesthesia for a patient undergoing nociception in surgery [10,51]. The low PSI value and the presence of SR were an indication of the inadvertently deep plane of anesthesia of the horses in the current study, which suggested that the anesthesia of these horses could have been lightened in order to reduce the depth of the anesthesia, had EEG been used to guide its monitoring, in addition to the traditional subjective anesthetic depth evaluations. The end-tidal isoflurane concentration showed an increase from S1 to S2. During S3, the SR value increased significantly from S2, indicating that the depth of anesthesia was far more excessive than in S2, despite the termination of isoflurane anesthesia at this time. Such an increase in the SR was mainly due to the absence of active surgical stimulation when the horses were being weaned from isoflurane anesthesia, but the cerebral cortex depression remained due to the accumulation of depressive effects from the previous excessive stages.

Regaining consciousness during the anesthetic recovery is a passive process, as well as a reversal of anesthetic induction [50,52]. Mechanisms involved in the anesthetic-induced loss of consciousness and regain of cerebral function have been studied, and much evidence supports the theory that general anesthetic drugs disrupt the communications of the neurocircuits that control the brainstem, thalamus, and cortex [10,14,15,28,37,38,51]. The basic functions that are controlled by the brainstem, including respiration, cardiovascular motor tone (blood pressure), swallowing, coughing, and eye reflexes, were used to score the subjective anesthetic depth in this study. These functions were lost soon after anesthetic induction, and gradually resumed during the recovery phase. It is vital to have a clear understanding of the anesthetic-induced neurophysiology and EEG data coupled with the clinical assessment in order to evaluate the overall brain state of an anesthetized patient.

Many of the depressive physiological events during S1 and S2 can be tied to the EEG changes, especially the changes in the brainwave from high-frequency low amplitude beta waves to low frequencies and high amplitude of δ, θ, and α waves (see Figure 4). The spectral edge frequency is the frequency below which 95% of the total EEG power is located [53]. Studies have shown that SEF_95_ decreased during isoflurane anesthesia compared with the awake state [53]. Unfortunately, we were unable to obtain the complete awake EEG of the horses in this study, thus could not compare the awake SEF_95_ with the anesthetized states. The mean value of the SEF_95_ during S1 and S2 were in the range of 8.19 to 8.65 Hz, indicating that the δ, θ, and α waves were dominant during these stages.

The presence of these dominant brainwaves in humans explains the neurocircuitry mechanisms of anesthetic-induced actions [10,14,15,51], which were observed in our study. For example, during S1, the anesthetic-induced brainstem depression abolished the swallowing reflex and spontaneous breathing function. The loss of these two functions via brainstem suppression and inhibition of reticular formation permitted an anesthetic state that allowed endotracheal tube intubation, retention, and respiration to be mechanically controlled by a ventilator without the horse breathing against it. This anesthetic-induced depressive state was maintained by isoflurane during S2 for the surgery. The horses not breathing (bucking) against the ventilator indicated that the transmission of the electrical impulses from the peripheral stretch receptors in the lungs, diaphragmatic, and intercostal muscles via the phrenic, glossopharyngeal, and vagus nerves were all inhibited and unable to communicate to the medulla oblongata respiratory center. The mean end-tidal isoflurane concentration during S2 was 1.3 ± 0.1% [54,55,56,57], which was a typical concentration used for maintaining equine surgery with a multimodal anesthetic protocol. Collectively, the presence of these slow-wave activities and the presence of burst suppression indicated that the functional connectivity of the thalamocortical loop (brainstem, thalamus, and cortex) was lost [15,52], and implies that these horses were profoundly unconscious. Furthermore, the presence of these waves also indicated that the sensory inputs from the lower part of the brain were blocked and could not be perceived by the cortex, and that profound antinociception and muscle relaxation were evident.

During S3, the SEF_95_ further decreased to mean values of 4.88 and 7.23 Hz from S2. This decrease in the SEF_95_ can be explained by the continuation of a profoundly depressed brain state despite the isoflurane being terminated. The lack of active surgical stimuli may have put these horses into a deeper cortical depression. This claim was supported by the significant increase in the SR values from S2 to S3. As the recovery progressed, the basic function of the brainstem was likely to be discordant from the cerebral cortex (i.e., consciousness) due to the termination of isoflurane and the metabolism of the induction drugs, which allowed the depressive effect of these anesthetics on the brainstem to be lifted. This then led to the return of swallowing and coughing reflexes, the resumption of spontaneous breathing, and an increase in muscle tone. Based on these signs, the horse was extubated in this study and entered S4. The increase in the mean PSI and EMG% during S3 aligned with these clinical observations.

The EMG monitoring capacity of the Sedline^®^ monitor is an added benefit to monitoring the frontal EEG in horses during anesthesia. The placement of the frontal electrodes allows detection of the frontal muscular activities (i.e., eye movements) involving the inter- and frontoscutularis, and the oculi ocularis muscles. Eye movements, such as active blinking and nystagmus, during surgery are reliable eye reflexes of a horse that is reacting to nociceptive stimuli or an inadequate plane of anesthesia. Consequently, during S3 and S4, the increase in the EMG% suggested the return of the motor function to these muscles, and the presence of nystagmus was a strong indicator that the horse was awakening. The significant increase in mean values of EMG from S1 and S2 to S3 and S4 support these clinical observations.

Xylazine is an alpha-2 agonist, similar to dexmedetomidine, and is commonly used to reduce the number of premature attempts to stand during equine recovery [58,59]. Xylazine acts on the locus coeruleus to cause sedation and can induce falsely low PSI values with a sleep-like state spindle pattern [10,18,30,60]. The administration of xylazine in this study resulted in a reduction in SEF_95_, but did not significantly change the PSI, SR, or EMG. These findings were different from the results of a human study [60] showing that dexmedetomidine CRI in sevoflurane anesthetized patients resulted in both SEF_95_ and PSI reduction, and enhanced the depth of sevoflurane anesthesia. The differences between our study and the human study may be due to the drug and route (IV bolus vs. CRI) of administration, the species, and the timing of drug administration on different inhalant anesthetics (isoflurane vs sevoflurane).

Equine recovery is known to be one of the most critical stages of the entire anesthesia course [61]. This is because one of the unique aspects of equine anesthesia, using isoflurane for maintenance, causes significant muscle relaxation during the early recovery stage, and premature attempts to stand before full control of muscles, posture, and gait balance are regained can put the horse at risk of limb fracture [62,63,64]. Furthermore, the actual brain state determining when a horse decides to stand and how it controls its own locomotion during the attempt to stand in recovery is currently unknown.

Studies in humans (and other species) on locomotion may shed some light in the brain state of how a horse decides to stand. Locomotion in humans is defined as the stages of a gait cycle to move from one place to another while maintaining balance and posture, showing complex coordination among the spinal central pattern generator, the brainstem reticular formation, the sensorimotor cortex, the basal ganglia, the basal forebrain, and the cerebellum, in order to properly control muscle tone, posture, and gait [65,66,67,68]. Furthermore, it has been shown that the motivation driving a locomotive episode involves internal or external needs, including the motor cortex, for a goal-driven or limbic system for emotion-driven or external motivation driven by environmental cues. Although we do not know exactly what motivates a horse to stand and what determines when a horse stands during anesthetic recovery, it is likely that a similar part of the brain structure reported in human locomotion may also play a role in supporting a horse to stand successfully [67].

In S4, the low mean PSI and the high SR value indicate that the central nervous system remained under the significant influence of anesthetics, despite the resumption of the brainstem’s basic function. These EEG data suggest that although the horses were extubated and resumed spontaneous breathing, the central nervous system and the brainstem that coordinated the muscle tone, posture, and gait balance remained depressed during this late recovery stage. We recognize the fact that the timing of when a horse decides to stand is far more complicated than what we have traditionally believed. It involves many parts of the brain that must work in concert to support the animal’s body weight and coordination, as it involves not only locomotion but also the motivation to drive them to stand successfully. Further studies are needed which focus on the use of EEG in understanding this area of physiology.

In conclusion, the result of this study showed that the EEG can be used to continuously monitor the brain state changes of an isoflurane-anesthetized horse with a multimodal anesthetic protocol.

## Figures and Tables

**Table 1:** The sex, breeds, age, and weight of the 12 horses enrolled in this study.

ID	Weight (kg)	Sex	Breed	Age (Years)
1	435	G	QH	10
2	439	F	QH	1
3	317	F	Standardbred	1
4	478	G	Thoroughbred	5
5	550	G	Thoroughbred	8
6	552	G	QH	7
7	543	M	QH	11
8	465	G	QH	6
9	396	G	Mix-breed	14
10	362	M	QH	1
11	454	F	Morgan	10
12	324	G	Thoroughbred	6

QH: Quarter Horse, G: Gelding, F: female, M: male (stallion).

**Table 2:** The types of surgeries, duration of anesthesia, and the recovery score of the horses enrolled in the study. For the recovery quality scores: 4 represents the worst and 1 represents the best.

ID	Type of Surgery	Total Anesthesia Time (h:min)	Prep-Time (h:min)	Surgery Time (h:min)	Recovery Time (h:min)	Recovery Score
1	Septic joint arthroscopy	1:35	00:40	00:51	1:20	2
2	Bilateral stifle arthroscopy	2:46	1:04	1:32	00:52	2
3	Fetlock arthroscopy	1:15	00:25	00:50	00:20	1
4	Hock arthroscopy	1:45	00:55	00:45	00:50	2
5	Hoof resection	1:21	00:40	00:32	00:30	3
6	Navicular bursoscopy	2:51	1:07	1:32	1:17	1
7	Cryptorchidectomy	2:34	00:40	00:51	00:52	3
8	Draining tract + mass removal	2:40	00:45	1:50	00:45	1
9	Heel bulb laceration repair	1:30	00:42	00:42	00:35	4
10	Castration	00:51	00:25	00:30	1:00	1
11	Ovariohysterectomy	2:11	00:29	1:36	1:10	2
12	Herniorrhaphy	2:30	00:53	1:45	1:32	1

**Table 3:** Processed EEG parameters during the 4 stages of anesthesia in the isoflurane-anesthetized horse. Stage 1—transition from induction to isoflurane anesthesia, stage 2—surgery during isoflurane maintenance, stage 3—early recovery before xylazine administration, and stage 4—late recovery. The 5-min average EEG data are analyzed.

		Stage 1	Stage 2	Stage 3	Stage 4
	Mean ± SD	1.3 ± 0.2	1.3 ± 0.1	1.1 ± 0.5	0.3 ± 0.1
ET_ISO_	Median (range)	1.4 (0.3–1.7)	1.3 (0.9–1.7)	1.4 (0.3–1.5)	0.3 (0.2–0.5)
	n	102	169	9	5
	Mean ± SD	24.8 ± 10.9	20.8 ± 2.7	30.0 ± 27.7	25.6 ± 14.9
PSI	Median (range)	22.1 (11.8–90.2)	21.2 (11.2–27.2)	22.3 (1.4–87.4)	22 (1.1–74.4)
	n	98	174	19	78
	Mean ± SD	5.5 ± 10.7	7.2 ± 12.1	32.7 ± 33.8	20.0 ± 24.7
SR	Median (range)	1.1 (0–52.7)	1.6 (0–54.9)	18.1 (0.4–93.2)	9.2 (0–95.4)
	n	102	179	19	79
	Mean ± SD	8.3 ± 2.7	8.2 ± 2.0	7.2 ± 4.8	4.6 ± 4.4
SEF L	Median (range)	8.4 (2.2–15.9)	8.4 (2.7–15.7)	6.2 (1.3–17.1)	2.8 (1.1–24.9)
	n	96	177	14	66
	Mean ± SD	8.7 ± 2.9	8.2 ± 2.2	4.9 ± 2.7	4.5 ± 2.7
SEF R	Median (range)	8.3 (4.0–17.1)	8.4 (1.1–14.1)	4.6 (1.4–11.2)	3.9 (1–13.8)
	n	96	175	14	70
	Mean ± SD	6.3 ± 9.3	6.0 ± 6.0	13.1 ± 19.1	11.0 ± 13.1
EMG	Median (range)	4.1 (0–59.3)	4.5 (0–22.2)	0.8 (0–55.9)	8.4 (0–65.5)
	n	102	179	19	79
	Mean ± SD	29.6 ± 23.5	23.5 ± 21.7	12.7 ± 13.2	13.4 ± 16
ART	Median (range)	25.8 (0.1–91.2)	17.2 (0–100)	7.1 (1.1–44.7)	7.0 (0–84)
	n	102	179	19	79

ET_ISO_: End-tidal isoflurane (%); PSI: Patient State Index; SR: Burst Suppression Ratio (%); SEF L/R: 95% of spectral edge frequency (Hz) on the R (right) or L (left) hemispheres; EMG: % of the electromyographic activity; ART: % of the artifact; “n” represent the total number of data points used for the data calculation.

## Data Availability

Data is contained within the article.

## Appendix A

**Table A1:** Subjective score categories and criteria used for evaluation of the depth of anesthesia score in the isoflurane-anesthetized horse. Modification from [19].

Anesthetic Plane	Score	Pupil Position/Size	Eye Reflex (P, Palpebral; C, Corneal)	Heart Rate/Blood Pressure
Too light	4	Central/ large or small	P: active, C: active	Normal or elevated /breathing against the ventilator
Medium	3	Ventromedial/ small or medium	P: depressed, C: mildly depressed	Normal or minimally depressed
Medium-deep	2	Central/ medium	P: depressed, C: depressed	Minimally to moderately depressed
Too deep	1	Central/ large	P: absent C: markedly depressed	Markedly depressed
